# Pro108Ser mutation of SARS-CoV-2 3CL^pro^ reduces the enzyme activity and ameliorates the clinical severity of COVID-19

**DOI:** 10.1038/s41598-022-05424-3

**Published:** 2022-01-25

**Authors:** Kodai Abe, Yasuaki Kabe, Susumu Uchiyama, Yuka W. Iwasaki, Hirotsugu Ishizu, Yoshifumi Uwamino, Toshiki Takenouchi, Shunsuke Uno, Makoto Ishii, Takahiro Maruno, Masanori Noda, Mitsuru Murata, Naoki Hasegawa, Hideyuki Saya, Yuko Kitagawa, Koichi Fukunaga, Masayuki Amagai, Haruhiko Siomi, Makoto Suematsu, Kenjiro Kosaki, Keio Donner Project

**Affiliations:** 1 Department of Surgery, Keio University School of Medicine, Tokyo, Japan; 2 Department of Biochemistry, Keio University School of Medicine, Tokyo, Japan; 3 Department of Biotechnology, Graduate School of Engineering, Osaka University, Osaka, Japan; 4 Exploratory Research Center On Life and Living Systems (ExCELLS), National Institutes of Natural Sciences, Okazaki, Japan; 5 Institute for Integrated Radiation and Nuclear Science, Kyoto University, Osaka, Japan; 6 Department of Molecular Biology, Keio University School of Medicine, Tokyo, Japan; 7 Division of Infection Diseases and Infection Control, Keio University Hospital, Tokyo, Japan; 8 Department of Infectious Diseases, Keio University School of Medicine, Tokyo, Japan; 9 Department of Pediatrics, Keio University School of Medicine, Tokyo, Japan; 10 Department of Internal Medicine, Keio University School of Medicine, Tokyo, Japan; 11 U-Medico, Inc., Osaka, Japan; 12 Department of Laboratory Medicine, Keio University School of Medicine, Tokyo, Japan; 13 Division of Gene Regulation, Institute for Advanced Medical Research, Keio University School of Medicine, Tokyo, Japan; 14 Department of Dermatology, Keio University School of Medicine, Tokyo, Japan; 15 Center for Medical Genetics, Keio University School of Medicine, 5 Shinanomachi, Shinjuku-ku, Tokyo, 160-8582 Japan

**Keywords:** Viral infection, Enzyme mechanisms

## Abstract

Recently, an international randomized controlled clinical trial showed that patients with SARS-CoV-2 infection treated orally with the 3-chymotrypsin-like protease (3CL^pro^) inhibitor PF-07321332 within three days of symptom onset showed an 89% lower risk of COVID-19-related hospital admission/ death from any cause as compared with the patients who received placebo. Lending support to this critically important result of the aforementioned trial, we demonstrated in our study that patients infected with a SARS-Cov-2 sub-lineage (B.1.1.284) carrying the Pro108Ser mutation in 3CL^pro^ tended to have a comparatively milder clinical course (i.e., a smaller proportion of patients required oxygen supplementation during the clinical course) than patients infected with the same sub-lineage of virus not carrying the mutation. Characterization of the mutant 3CL^pro^ revealed that the Kcat/Km of the 3CL^pro^ enzyme containing Ser108 was 58% lower than that of Pro108 3CL^pro^. Hydrogen/deuterium-exchange mass spectrometry (HDX-MS) revealed that the reduced activity was associated with structural perturbation surrounding the substrate-binding region of the enzyme, which is positioned behind and distant from the 108th amino acid residue. Our findings of the attenuated clinical course of COVID-19 in patients infected with SARS-CoV-2 strains with reduced 3CL^pro^ enzymatic activity greatly endorses the promising result of the aforementioned clinical trial of the 3CL^pro^ inhibitor.

## Introduction

A recent international randomized controlled clinical trial showed that the SARS-CoV-2 3-chymotrypsin-like protease (3CL^pro^) inhibitor PF-07321332 is orally bioavailable and highly effective in reducing the clinical severity of COVID-19. Patients who received oral treatment with this inhibitor within three days of symptom onset showed an 89% lower risk of COVID-19-related hospital admission/death from any cause as compared with patients who received placebo^1^. Regulatory agencies of various countries are ready to initiate review of PF-07321332 for pharmaceutical approval. Consistent with the proposed efficacy of the 3CL^pro^ inhibitor, patients infected with a sub-lineage of SARS-CoV-2 carrying a loss-of-function mutation in 3CL^pro^ appear to show a less severe clinical course.

The SARS-CoV-2 virus has relatively limited fidelity for genome replication and its genome accumulates point mutations at an average of two nucleotides per month (GISAIDs: http://www.gisaid.org/); various parts of the genome can be subject to mutations^2,3^. Our institution, Keio University Hospital, has a catchment area that includes the Tokyo Metropolitan area and surrounding prefectures and we have been performing whole viral genome sequencing of SARS-CoV-2 in COVID-19 patients since March 2020, with the aim of characterizing healthcare-associated infections rapidly and effectively^4^. Through molecular surveillance of patients infected in the Tokyo Metropolitan area, we found that in the summer of 2020, a sub-lineage of the virus with non-synonymous Pro108Ser mutation in the 3CL^pro^ protein became relatively dominant. During the same period, the relative proportion of seriously ill patients and the mortality rate also decreased (https://www.mhlw.go.jp/stf/covid-19/kokunainohasseijoukyou.html#h2_1)^5^.

We hypothesized in our clinical study that the patient group infected with the SARS-Cov-2 sub-lineage (B.1.1.284) carrying the Pro108Ser mutation in 3CL^pro^ tended to have a comparatively milder clinical course (i.e., a smaller proportion of patients required oxygen supplementation during the clinical course) than the patient group infected with the same sub-lineage not carrying the mutation. In parallel with the clinical study, we conducted in vitro experiments to characterize Pro108Ser mutant 3CL^pro^ enzyme from a biochemical standpoint.

## Results

### Viral genome sequence analysis

A mean of 14.5 (± 4.0) mutations separated the presently reported lineage from the founding Wuhan haplotype (the central haplotype of Clade A). None of the strains had truncating mutations such as frameshift or nonsense mutations. The number counts of non-synonymous mutations among the strains varied from 2 to 12 (mean, 7.8 ± 2.0), compared with the Wuhan reference strain.

### Clinical background of COVID-19 patients

The clinical characteristics of the 179 patients are shown in Supplementary Table 1. Forty-five patients (25.1%) required supplemental oxygen, and fifteen (8.4%) developed acute respiratory distress syndrome; seven of these fifteen patients died.

### Phylogenic tree analysis in our study cohort

**Figure 1:**
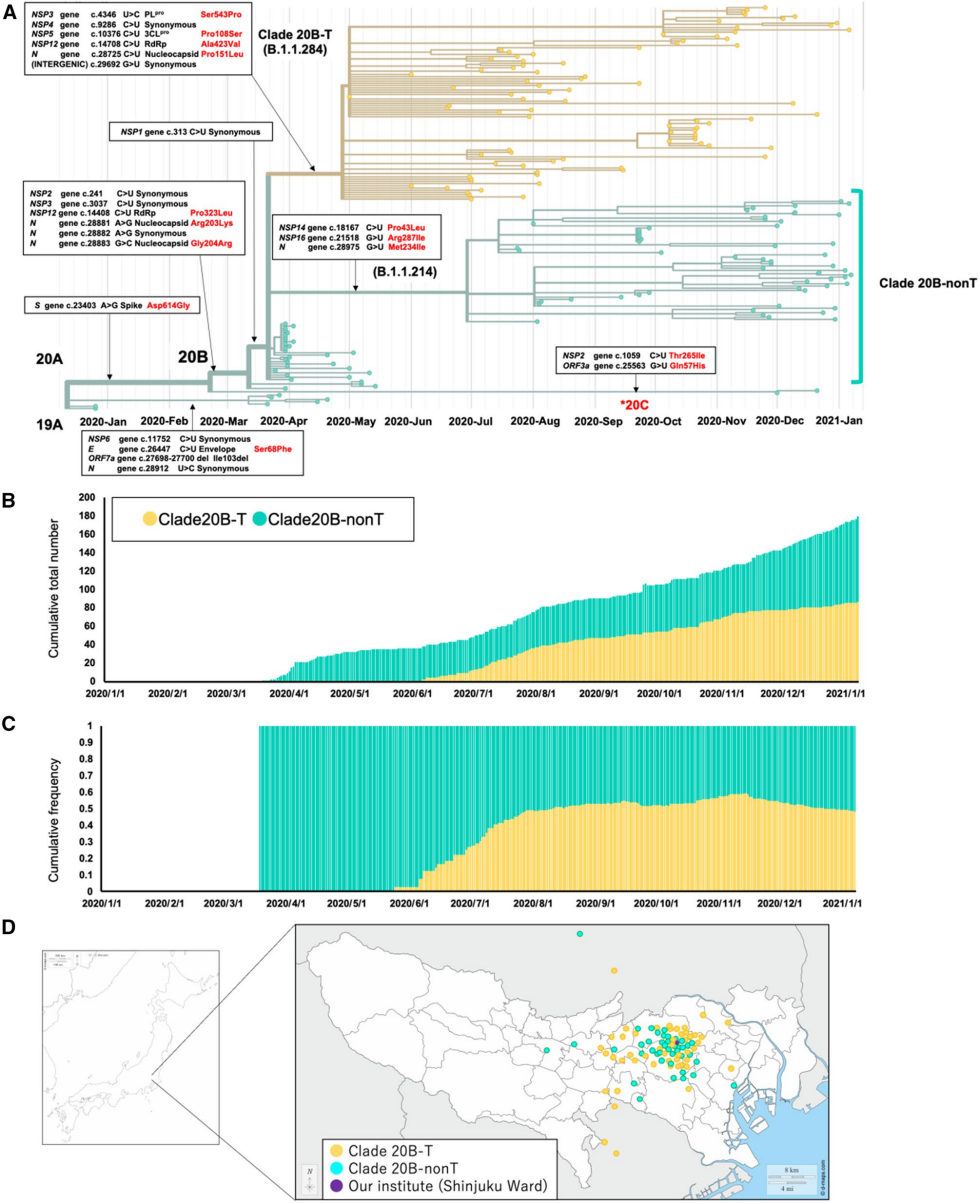
Phylogenic tree analysis, temporal trends, and spatial distribution around Keio University Hospital (purple dot in **d**) showed consistent increase of a strain with a unique haplotype. (**a**) Connection of Keio Strains (right, N = 179) to the clades defined by GISAID was described in the time-resolved phylogenic tree. Yellow branches represent the predominant Clade 20B-T (B.1.1.284) in Keio University Hospital which had the six additional mutations compared with the remaining strains Clade 20B-nonT of Clade 20B. *These two strains (Clade 20C) indicate the intrusion from foreign countries. (**b**) Temporal trends of the number of patients of Clade 20B-T (B.1.1.284) and Clade 20B-nonT at our institute. (**c**) The cumulative frequency of Clade 20B-T (B.1.1.284) at our institute. (**d**) The suspected location of infection of individuals from Clade 20B-T (B.1.1.284) scattered over the Tokyo Metropolitan area and its neighboring prefectures. These maps were cited from the following sources; https://d-maps.com/continent.php?num_con=16&lang=ja, COVID-19, coronavirus disease 2019; NSP, Non-structural polyprotein; PL^pro^, papain-like proteinase; 3CL^pro^, 3 chymotrypsin-like protease; RdRp, RNA dependent RNA polymerase; ORF, open reading frame.

We investigated whether any of the phylogenic clade containing non-synonymous mutations contributed to a milder clinical course. The overall genetic diversity was relatively low, presumably because effective international border restrictions and successful quarantine efforts were in place. A divergent tree analysis of the whole viral genome sequences and classification at Keio University Hospital (N = 179) according to the internationally recommended nomenclature showed that most patients (i.e., 172 [96.1%]) patients had strains derived from Clade 20B (Fig. 1a)^6^. The remaining 5 and 2 patients in our cohort study belonged to Clade 19A and 20C, respectively^7,8^; these patients were therefore excluded from further study. Patients from Clade 20B were additionally divided into two subgroups by defining each subgroup as containing patients who had strains with no more than 5 nucleotide differences. The first subgroup was designated as the Subclade 20B-T (Pangolin lineage B.1.1.284^9^; N = 87 [50.6%]), which had the basic haplotype of Clade 20B with the addition of 6 single nucleotide mutations: c.4346 U > C, c.9286 C > U, c.10376 C > U, c.14708 C > U, c.28725 C > U and c.29692 C > U (Fig. 1a, yellow). The transmission of this infection in the central downtown area led to this strain spreading to the rest of Japan in June 2020. Of the six mutations, four were non-synonymous: c.4346 U > C (Ser543Pro in papain-like protease [PL^pro^]), c.10376 C > U (Pro108Ser in 3CL^pro^), c.14708 C > U (Ala423Val in RNA-dependent RNA polymerase [RdRp]), and c.28725 C > U (Pro151Leu in nucleocapsid protein); the remaining two other mutations did not affect the amino acid translation of the viral proteins. The second subgroup was designated as Clade 20B-nonT (N = 85 [49.4%]), which showed the haplotype of Clade 20B and was defined by the presence of seven possible mutations separating the lineage from the founding Wuhan haplotype, although each case had fewer than five single nucleotide mutations. Analyses of the cumulative total number and frequency curve showed that the relative fraction of Clade 20B-T (B.1.1.284) increased during the time frame of this study (Fig. 1b, c). Mapping of the suspected geographic locations where the infection of individual patients was likely to have occurred indicated that the patients with Clade 20B-T (B.1.1.284) or Clade 20B-nonT were infected in the Tokyo Metropolitan area and its neighboring prefectures (Fig. 1d). This observation, together with a lack of patients with strains belonging to other clades (except for the 5 and 2 patients with strains belonging to Clade 19A and 20C, respectively) suggested that Clade 20B and its variation Clade 20B-T (B.1.1.284) were the predominant strains in the Tokyo Metropolitan area from June to October 2020. From November 2020 to January 2021, the number of patients with Clade 20B-T (B.1.1.284) decreasing, while the number of patients with B.1.1.214 among Clade 20B, which first emerged in July 2020, simultaneously increased. The B.1.1.214 lineage is characterized by the addition of 3 single nucleotide mutations with the basic haplotype of Clade 20B: c.18167 C > U (Pro43Leu in 3′–5′ endonuclease), c.21518 G > U (Arg287Ile in 2′-O ribose methyltransferase), and c.28975 G > U (Met234Ile in nucleocapsid protein).

### Clade 20B-T patients exhibited milder clinical courses

A comparison of the clinical characteristics of the patients with Clade 20B-T (B.1.1.284; N = 87) and those of the patients with Clade 20B-nonT (N = 85) is shown in Table 1. Age, sex, symptoms at admission, and outcome did not differ significantly between the two main groups. However, the numbers of patients who required oxygen supplementation and methylprednisolone treatment were significantly lower among the patients with Clade 20B-T (B.1.1.284) than among those with Clade 20B-nonT (oxygen supplementation, 14.9% vs. 30.6%, *p* value = 0.011; methylprednisolone treatment, 11.5% vs. 20.0%, *p *value = 0.045). An exact logistic regression analysis showed that patients with Clade 20B-T (B.1.1.284) had a lower odds ratio for developing hypoxia requiring supplemental oxygen, compared with those with Clade 20B-nonT (adjusted odds ratio, 0.35 [95% CI 0.14–0.88], *p *value = 0.026; Table 2) after adjustments for age group (< 65 years or ≧ 65 years), sex (male or female), temporal trends (from March to October 2020 or from November 2020 to January 2021) and Charlson Comorbidity Index group (0 or ≧1). A comparison of patients infected with B.1.1.214 and those infected with B.1.1.284 revealed that patients infected with B.1.1.214 tended to require oxygen supplementation (odds ratio, 2.32 [95% CI, 0.94–5.84], *p *value = 0.055).

**Table 1:** Comparison of clinical features between Clade 20B-T and Clade 20B-nonT.

N = 172	Clade 20B-T [B.1.1.284] (N = 87)	*Clade 20B-nonT (N = 85)	*p* value
Mean age (years old)	43.0 ± 17.6	45.6 ± 19.4	0.353
Sex (male/female)	57 / 30	56 / 29	1.000
Charlson Comorbidity Index (≧1)	29 (33.3%)	38 (44.7%)	0.159
**Symptoms at admission**			
Cough	40 (46.0%)	44 (51.8%)	0.756
Dysosmia	12 (13.8%)	13 (15.3%)	1.000
Dysgeusia	10 (11.5%)	15 (17.6%)	0.388
Fever (≧ 37·5 °C)	51 (58.6%)	52 (61.2%)	0.432
Sepsis	0	1 (1.2%)	0.494
Acute respiratory distress syndrome	3 (3.4%)	8 (9.4%)	0.130
**Treatment**			
Oxygen supplementation	13 (14.9%)	26 (30.6%)	0.011
Methylprednisolone treatment	10 (11.5%)	20 (23.5%)	0.045
Ventilator usage	2 (2.3%)	6 (7.1%)	0.166
Intensive care unit admission	4 (4.6%)	8 (9.4%)	0.245
Death	2 (2.3%)	2 (2.4%)	1.000

*Clade 20B-nonT includes various strains defined by Pangolin lineages; B.1.1.214, B.1.1.285, B.1.1.162, and B.1.1.29.

**Table 2:** Logistic regression analysis of candidate predictors for requiring supplemental oxygen.

	Univariate model		Multivariate model	
	OR (95% CI)	*p* value	Adjusted OR (95% CI)	*p* value
**Age, years**				
< 65	1 (ref)	–	1 (ref)	–
≧ 65	9.81 (3.90–25.87)	< 0.001	2.52 (0.91–7.02)	0.076
**Sex**				
Female	1 (ref)	–	–	–
Male	1.77 (0.76–4.41)	0.186	–	–
**Charlson Comorbidity Index**				
0	1 (ref)	–	1 (ref)	–
≧ 1	21.40 (7.51–76.01)	< 0.001	14.11 (4.53–43.99)	< 0.001
**Temporal trend**				
March to October 2020	1 [ref]	–	–	–
November 2020 to January 2021	1.69 (0.76–3.73)	0.180	–	–
**Infection**				
Clade 20B-nonT	1 (ref)	–	1 (ref)	–
Clade 20B-T [B.1.1.284]	0.38 (0.16–0.84)	0.011	0.35 (0.14–0.88)	0.026

OR, odds ratio; CI, cofidence interval.

### Molecular evolutionary characterization of four non-synonymous mutations unique to Clade 20B-T

**Figure 2:**
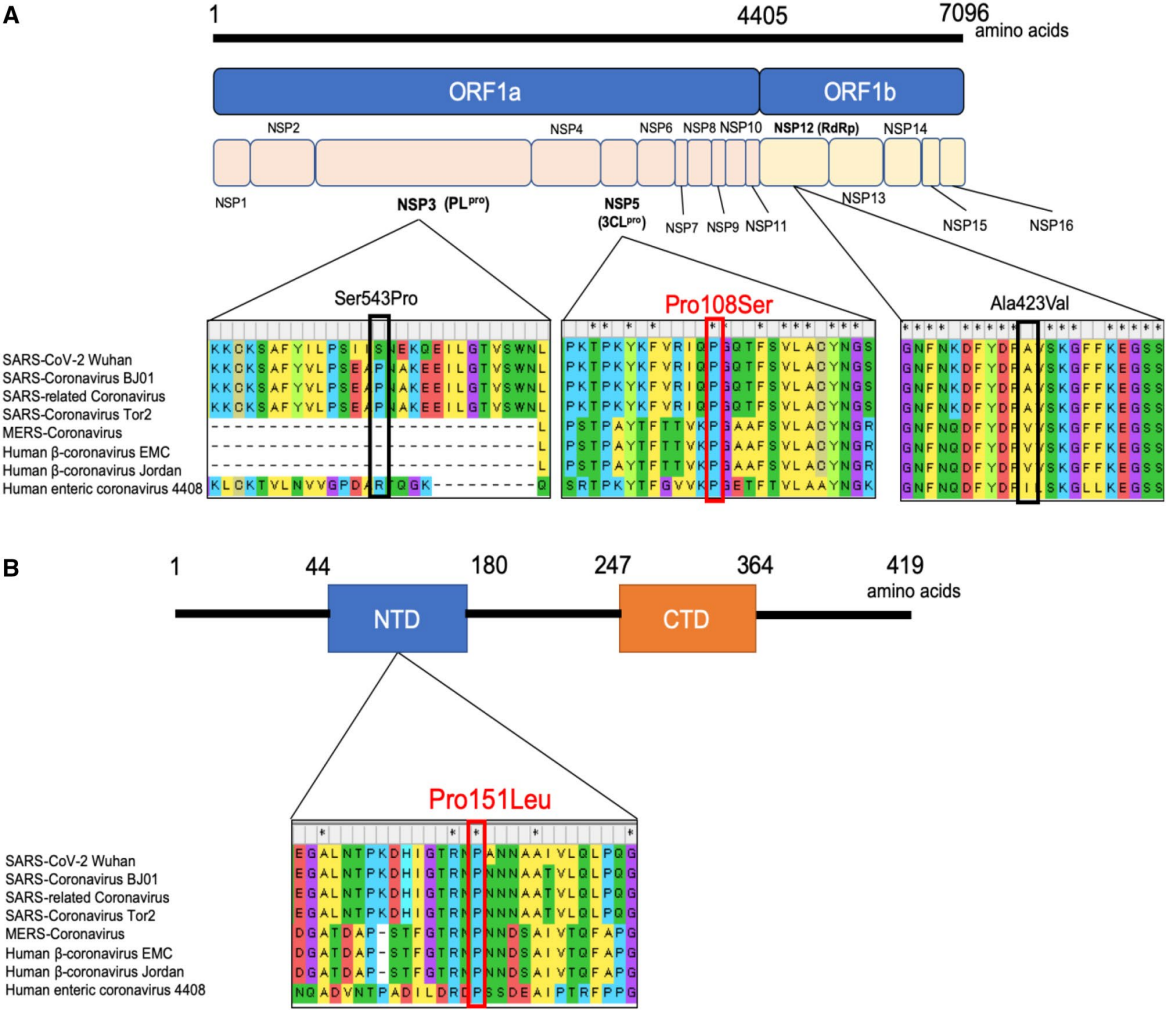
Multiple amino acid sequence alignments of various β-coronaviruses and locations of mutated amino acid residues in Clade 20B-T (B.1.1.284). (**a**) The structure of the genomic region that encodes nonstructural polyproteins of SARS-CoV-2. Multiple sequence alignments homologous proteins of 7 β-coronaviruses at and around 3 non-synonymous mutations using Molecular Evolutionary Genetic Analysis software (MEGA, https://www.megasoftware.net/) and Microsoft power point 2019: Ser543Pro in the PL^pro^, Pro108Ser in the 3CL^pro^, and Ala423Val in the RdRp. (**b**) The structure of the genomic region that encodes nucleocapsid protein of SARS-CoV-2. Multiple sequence alignments homologous proteins of 7 β-coronaviruses at and around the non-synonymous mutation Pro151Leu in the nucleocapsid protein using Molecular Evolutionary Genetic Analysis software (MEGA, https://www.megasoftware.net/) and Microsoft power point 2019. SARS-CoV-2, severe acute respiratory syndrome coronavirus 2. ORF, open reading frame; NSP, nonstructural protein; PL^pro^, papain-like protease; 3CL^pro^, 3 chymotrypsin-like protease; RdRp, RNA-dependent RNA polymerase; SARS, severe acute respiratory syndrome; MERS, middle east respiratory syndrome; NTD, N-terminal domain; CTD, C-terminal domain; COVID-19, coronavirus disease 2019; MEGA, Molecular Evolutionary Genetics Analysis.

We used molecular evolutionary analyses to decipher which of the four non-synonymous mutations that characterize the Clade 20B-T haplotype contributed to the milder clinical course. Studies of the conservation of the amino acid residues around the non-synonymous mutations in Clade 20B-T indicated that residues at and around Pro108Ser in the 3CL^pro^ (NSP5) and those at and around Pro151Leu in the nucleocapsid protein were highly conserved among β-coronaviruses (Fig. 2a, b). By contrast, amino acid residues at and around Ser543Pro in the PL^pro^ (NSP3) and Ala423Val in RNA-dependent RNA polymerase (RdRp, NSP12) were only weakly conserved (Fig. 2a). On the other hand, the serine in the PL^pro^ at residue 543 and the Ala at residue 423 in RdRp were substituted with proline and valine in some β-coronaviruses; both of these mutations were observed in Clade 20B-T, suggesting that Ser543Pro in the PL^pro^ and Ala423Val in RdRp are likely to be functionally neutral. PROVEAN predicted that these 2 mutations were not deleterious. Pro108Ser in the 3CL^pro^ and Pro151Leu in the nucleocapsid protein can therefore be considered as plausible candidate amino acid mutations in Clade 20B-T (B.1.1.284) that are functionally relevant and may explain the milder clinical course observed among patients infected with Clade 20B.

### Phylogenic tree analysis of sequences in Japan registered by GISAID

**Figure 3:**
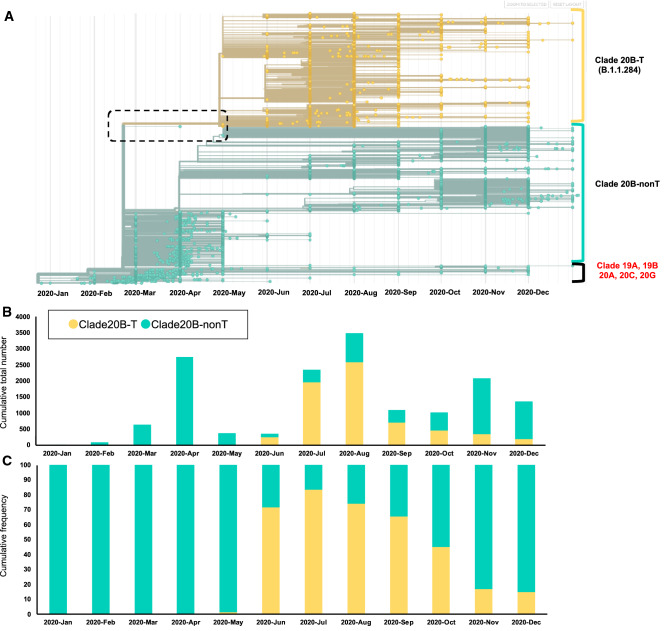
Phylogenic tree analysis, temporal trends in Japan registered by GISAID database. (**a**) Most of the Japanese strains were derived from National Institute of Infectious Diseases (NIID) submitted on 10th January 2021 in GISAID excluding airport quarantine, but were not specified by towns/cities or precise obtaining dates (obtaining month only). Therefore, we designated all the NIID data for the first day of the month (i.e., 2020/4 → 2020/4/1). A magnified view of the dotted square is shown in Supplementary Fig. 1. (**b**) Temporal trends of the number of patients of Clade 20B-T (B.1.1.284) and Clade 20B-nonT in Japan. (**c**) The cumulative frequency of Clade 20B-T (B.1.1.284) in Japan. COVID-19, coronavirus disease 2019; NSP, Non-structural polyprotein; PL^pro^, papain-like proteinase; 3CL^pro^, 3 chymotrypsin-like protease; RdRp, RNA dependent RNA polymerase; ORF, open reading frame.

A phylogenic analysis of the whole viral genome sequences in Japan that have been registered by GISAID (N = 16,134) showed that most patients (N = 15,367; 95.2%) had strains derived from Clade 20B, and Clades 20B-T (B.1.1.284) (N = 6770) and B.1.1.214 (N = 5051) derived from Clade 20B turned out to be the predominant strains since November 2020; these findings were compatible with those of the present study cohort (Fig. 3a)^6^. Analyses of the cumulative total number and frequency curve showed that the relative fraction of Clade 20B-T (B.1.1.284) increased between June and October 2020; however, since November 2020, the relative fraction of Clade 20B-T (B.1.1.284) has been decreasing over time, mainly due to the increase in B.1.1.214 (Fig. 3b, c).

Based on the results described above, because 3CL^pro^ has been well characterized as a critical function for viral replication by biochemical and pharmacological analyses^10,11^, and because the phylogenic tree analysis in Japan showed that the Pro151Leu mutation in nucleocapsid protein occurred earlier than the Pro108Ser mutation (Supplementary Fig. 1), we focused on the function-structure relationship of the Pro108Ser mutant of 3CL^pro^ for further investigation.

### P108S 3CL^pro^ reduces the catalytic activity and attenuates the sensitivity to GC376

**Figure 4:**
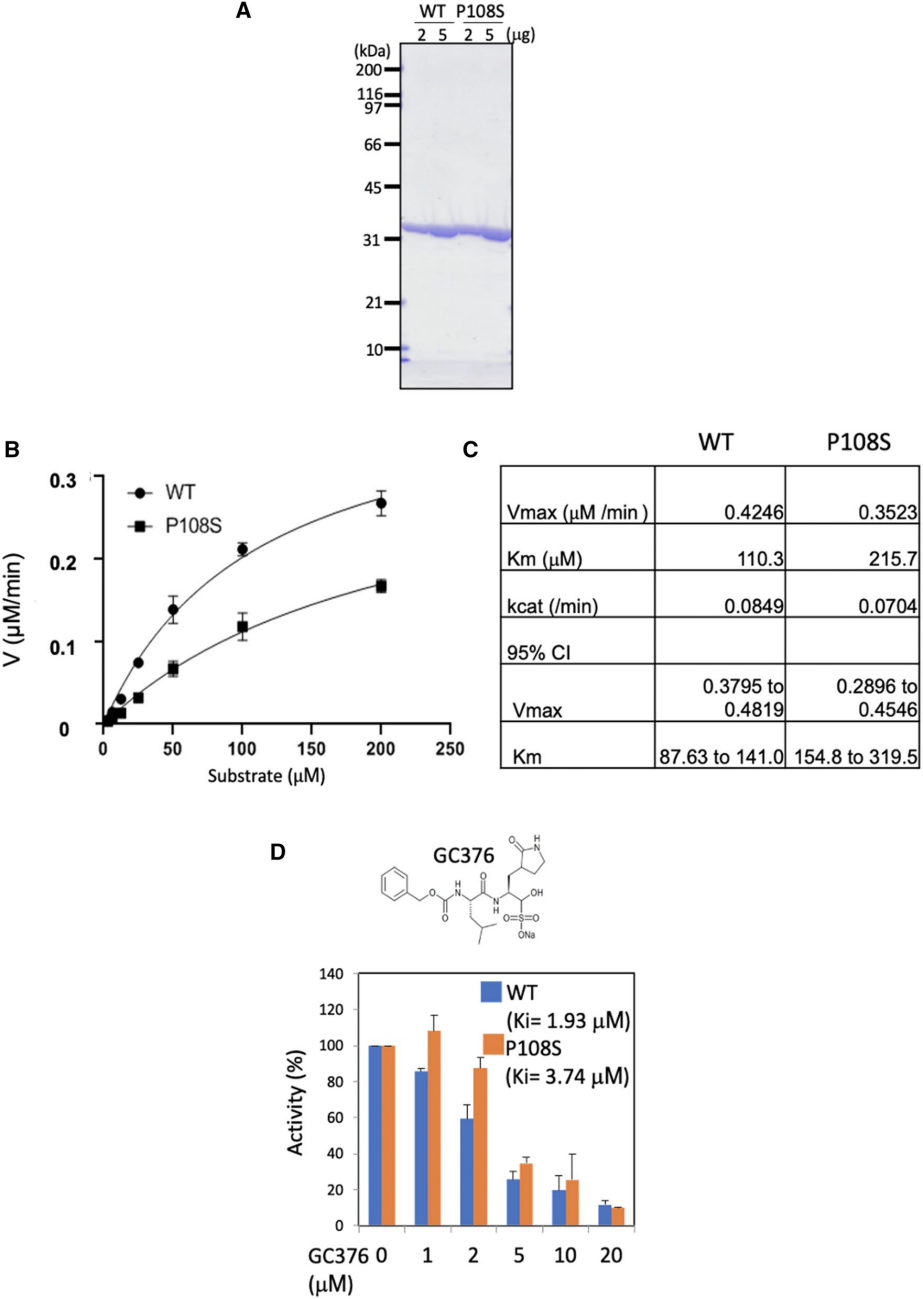
SARS-CoV-2 3CL^pro^ P108S is declined its enzymatic activity by structural alteration. (**a**) Recombinant WT or P108S of SARS-CoV2 3CL^pro^ were analysed with SDS-PAGE visualizing using CBB staining. (**b**) The enzymatic activities of SARS-CoV2 3CL^pro^ WT (circle) and P108S (square) were determined using a FRET-based substrate with the cleavage site of SARS CoV-2 3CL^pro^ (Dabcyl-KTSAVLQ↓SGFRKME-Edans). Error bars show mean ± SD (n = 3). (**c**) The kinetic parameters of enzyme activity of 3CL^pro^ WT and P108S were determined using GraphPad Prism 8 software by initial rate measurement of the substrate cleavage. The Kcat/Km value of the P108S mutant enzyme was 42% of that of the WT enzyme, showing 58% reduction. CI indicates 95% confidence interval. (**d**) Inhibitory activities of SARS-CoV2 3CL^pro^ WT and P108S by GC376 were analyzed using a FRET-based cleavage assay. The graph shows the relative enzymatic activity. The inhibitory constant (Ki) was calculated using GraphPad Prism 8 software. Error bars show mean ± SD (n = 3). SARS-CoV-2, severe acute respiratory syndrome coronavirus 2; 3CL^pro^, 3 chymotrypsin-like protease; WT, Wuhan-strain type; P108S, Pro108Ser-strain type; SDS-PAGE, Sodium dodecyl sulfate–Polyacrylamide gel electrophoresis; CBB, Coomassie Brilliant Blue; FRET, fluorescence resonance energy transfer.

We prepared recombinant proteins of WT and P108S of SARS-CoV-2 3CL^pro^ to determine their enzymatic activities using a fluorescence-based cleavage assay (Fig. 4a)^11^. The uncropped gel image of Fig. 4 was shown in Supplementary information. The enzymatic activity of the P108S was significantly suppressed, compared with that of the WT (Fig. 4b). The Km value of P108S (215.7 μmol/l) was lower than that of the WT (110.3 μmol/l), and the activity also decreased by 58%, as determined by a comparison of the Kcat/Km values for the WT and P108S 3CL^pro^ enzymes (Fig. 4c). These results suggest that the P108S mutation interferes with the ability of the enzyme to allow substrate binding.

We further examined the sensitivity of the P108S mutant against a competitive 3CL^pro^ inhibitor GC376. Recently, a feline infectious peritonitis virus (FIPV) inhibitor GC376 has been reported to block the SARS-CoV-2 3CL^pro^ activity by binding to the substrate-binding pocket^12,13^. The enzymatic activity of the WT protein was potently inhibited (Ki = 1.93 μmol/l) by GC376. On the other hand, the inhibitory effect of GC376 on the P108S mutant was decreased (Ki = 3.74 μmol/l; Fig. 4d).

**Figure 5:**
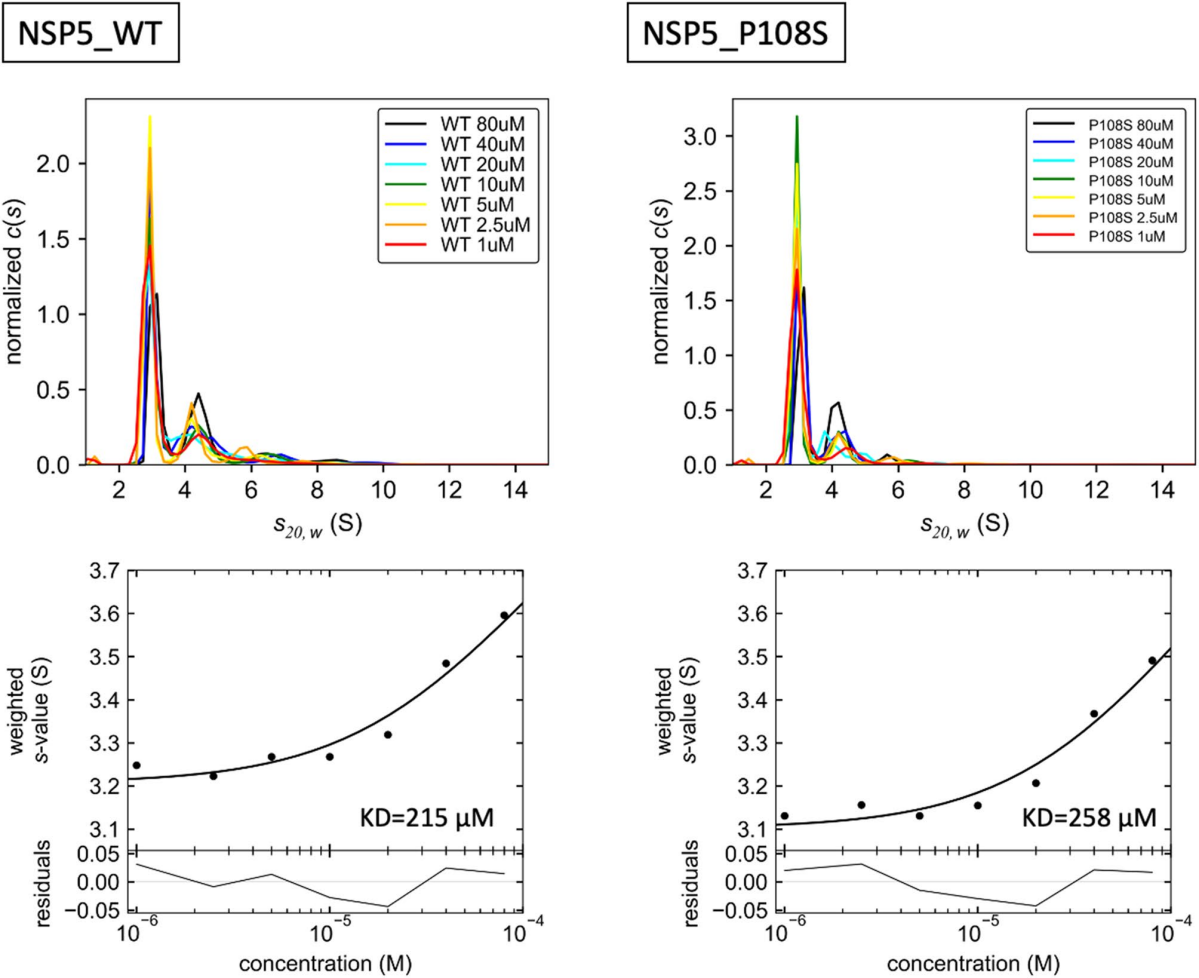
The analysis of 3CL^pro^ structure sequence by SV-AUC. These experiments were performed using the Optima AUC. The collected data were analyzed using continuous c(s) distribution model implemented in program SEDFIT (version 16.2b). The concentration dependence of the weight-average sedimentation coefficient was fitted to the monomer–dimer self-association model implemented in program SEDPHAT (version 15.2b). 3CL^pro^, 3 chymotrypsin-like protease; SV-AUC, sedimentation velocity analytical ultracentrifugation.

**Figure 6:**
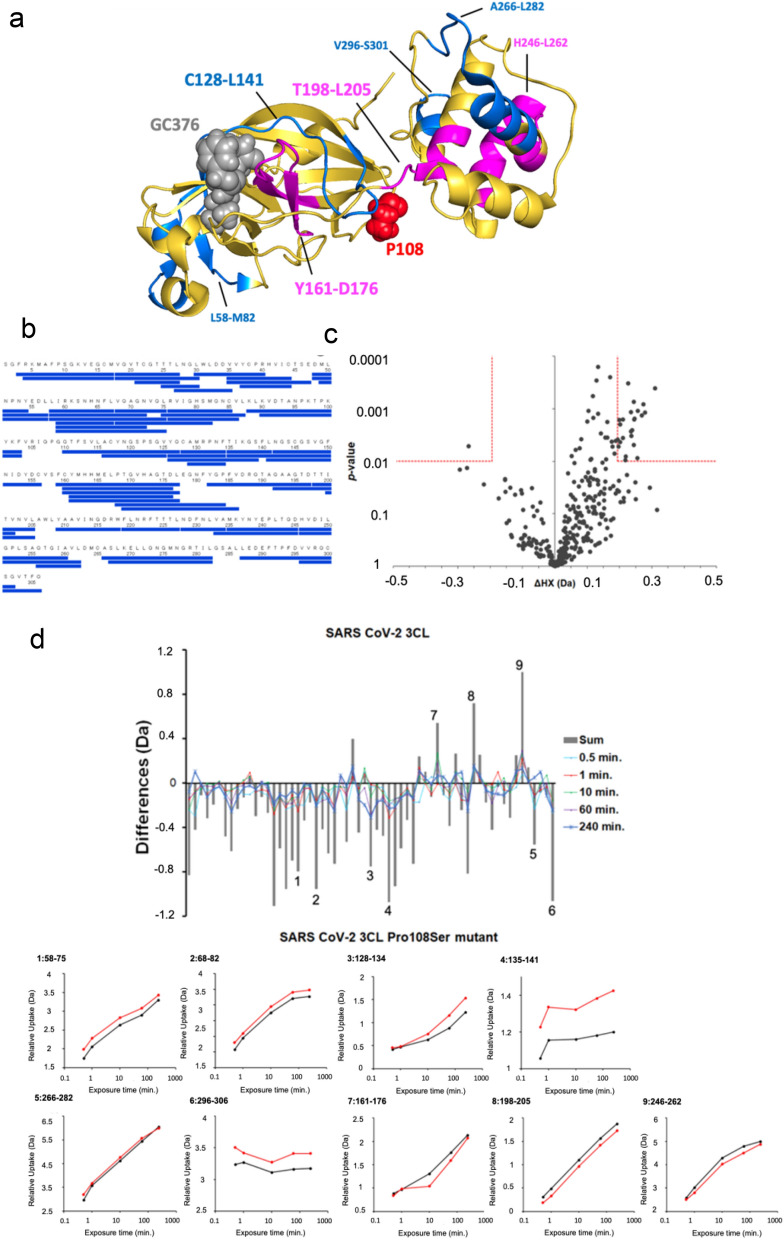
HDX-MS results of SARS CoV-2 3CL^pro^ WT and P108S. (**a**) Structurally influenced regions accompanied by a single mutation at 108th amino acid from proline to serine. HDX-MS showed more protected regions (magenta) and more exposed regions (cyan) in SARS-CoV-2 3CL^pro^ Pro108Ser mutant compared to SARS-CoV-2 3CL^pro^. Mutation of proline to serine at 108th amino acid induces structural alternation at the regions from C128 to L141 and from Y161 to D176, where C128-L141 is sandwiched between P108 and Y161-D176 which is located at the substrate binding region. (**b**) The coverage map of identified peptides in SARS-CoV-2 3CL^pro^. (**c**) Volcano plots of observed delta HDX values and *p *values calculated from Welch’s t-test for SARS-CoV-2 3CL WT. Red lines showed the horizontal *p *value and the vertical delta HDX values for the significant criteria. (**d**) Differential plots of deuterium uptake degrees of peptides, showing time courses, along with their summational results (gray bar). Deuterium uptake curves for the peptides showing significant differences between WT (black) and P108S (red) proteins were presented. SARS-CoV-2, severe acute respiratory syndrome coronavirus 2; 3CL^pro^, 3 chymotrypsin-like protease; WT, Wuhan-strain type; P108S, Pro108Ser mutant. HDX-MS, Hydrogen/deuterium exchange mass spectrometry.

Since previous studies of SARS-CoV 3CL^pro^ have indicated that the dimerization of 3CL^pro^ activates its enzymatic activity^14,15^, we analyzed the dimeric states of SARS-CoV-2 3CL^pro^ WT and P108S using sedimentation velocity analytical ultracentrifugation (SV-AUC). The results indicated comparable concentration dependencies of the weight averaged s-value (Fig. 5), indicating that the values of the monomer–dimer dissociation constants were comparable between WT and P108S mutant proteins within the given concentration ranges. An analysis using circular dichroism (CD) spectroscopy showed no discernible differences in the secondary and tertiary structures between the two proteins (Supplementary Fig. 2). These results led us to further examine alterations in the microenvironments in and around the substrate-binding site of 3CL^pro^. HDX-MS enabled the detection of structural perturbations around the substrate-binding pocket including C128-L141 close to P108 and Y161-D176 (Fig. 6a–d), suggesting that the P108S mutation perturbs the pocket that is behind and distant from the mutation.

## Discussion

Patients in this study cohort who were infected with the virus sub-lineage that carried the Pro108Ser of 3CL^pro^ (N = 87) (which is the main protease in the virus that cleaves the viral polyproteins into individual proteins that exert viral functions)^16^ tended to have a milder disease course than those infected with a viral sub-lineage that did not carry this mutation of 3CL^pro^ (N = 85). An in vitro enzymatic assay of recombinant Pro108Ser showed that the Kcat/Km value of the mutant 3CL^pro^ containing Ser108 was 58% lower as compared with that of 3CL^pro^ containing Pro108.

HDX-MS revealed that the substrate binding site is locally impacted by the mutation, leading to a reduced substrate binding affinity, even though the Pro108Ser did not affect the overall structure or association site the SV-AUC and CD spectroscopy sites. Thus, despite the distance between the mutation site and the substrate-biding site, Pro108Ser appears to play a critical role in the reduced enzyme activity and may abrogate both the replication potency and pathogenicity of the virus. While the mechanisms by which the proline substitution reduces the 3CL^pro^ enzyme activity remain unknown, the notion that the proline residue in the protein both disfavors helix formation and confers local rigidity to the polypeptide chain^17^, as demonstrated by HDX-MS, suggests that structural perturbation surrounding the substrate-binding region, which is positioned behind and distal to the 108th amino-acid residue of 3CL^pro^, plays a critical role in the decline of the enzyme function. While the 3CL^pro^-mutant SARS-CoV-2 disappeared by January 2021 in Japan, we believe that any variants possessing the P108 3CL^pro^ mutation that might emerge in the future would be less pathogenic, but also less sensitive to small-molecule compounds targeting the substrate-binding sites of this enzyme that are under development. Further investigation based on global surveillance of new variants would be needed.

During the COVID-19 pandemic, many countries have seen the spread of SARS-CoV-2 variants belonging to multiple different clades^18,19^, but in Japan in summer of 2020, variants belonging mainly to Clade 20B have accounted for most of the viral spread throughout the country. This study has serendipitously served as experiment of nature examining the roles of 3CL^pro^ activity in the virus in the presence of minimally divergent spread of different variants, presumably because of the successful quarantine measures in Japan since March 2020^20,21^.

The viral-producing proteins, such as the main protease or RNA polymerase, have been considered as therapeutic targets for coronaviruses, and various therapeutic agents and vaccines have been developed^22^. Apart from human coronavirus infections, GC376 (a bisulfite prodrug) has been shown to be effective against FIPV, which belongs to the α-coronavirus family. The administration of GC376 is associated with a high rate of disease remission and no significant adverse effects when used against FIPV^23^. GC376 is known to be a broad-spectrum protease inhibitor, and is converted to the peptide aldehyde GC373 and interacts covalently with the catalytic cysteine of coronavirus 3CL^pro^. In the presence of GC373, the N terminal finger (Ser1, Phe140, and Glu166) of 3CL^pro^ is strongly hydrogen-bonded with the active site (His41, Cys145) of the 3CL^pro^. Hence, GC373 stabilizes the substrate-binding site (Glu166), inhibiting the dimerization of 3CL^pro^^24–26^.

PF-07321332, an orally bioavailable derivative of GC373, was recently shown to exhibit antiviral activity both in vivo and in vitro^27^. PF-07321332 inhibited SARS-Cov-2-induced cytopathic effect in VeroE6 cells enriched with ACE2, and inhibited SARS-CoV-2 replication in A549 cells expressing ACE2^27,28^. PF-07321332 was also shown to be effective in a mouse-adapted SARS-CoV-2 MA10 model^29^. Based on these preclinical data, an international randomized controlled clinical trial named “Evaluation of Protease Inhibition for COVID-19 (EPIC)” was initiated. An interim analysis of the EPIC phase 2/3 trial conducted in high-risk patients showed that the inhibitor drug is very effective in reducing hospitalization/death.

The clinical efficacy data will be submitted to the U.S. FDA for Emergency Use Authorization. Phase 2/3 studies of the efficacy of the enzyme inhibitor in standard-risk patients and as post exposure prophylaxis are under way. Our documentation of an attenuated clinical course in patients infected with the mutant 3CL^pro^ endorses the notion that 3CL^pro^ inhibitors might be effective in reducing the severity of COVID-19 in humans. Cell-based cytotoxic and replication assays, similar to the ones performed in preclinical studies of PF-07321332, would further validate the direct linkage between reduced viral replication and the P108S mutation in 3CL^pro^ of SARS-CoV-2.

## Methods

### Study design and patients

A total of 311 patients who had been diagnosed as having COVID-19 between March 17, 2020, and January 7, 2021, based on the basis of reverse transcription polymerase chain reaction (RT-PCR) results at Keio University Hospital were enrolled. Of these 311 patients, 229 (73.6%) underwent whole viral genome sequencing. Of these, 50 patients with only partial genome sequences resulting from insufficient PCR amplification were excluded, leaving 179 patients for inclusion in the present analysis (Supplementary Fig. 3). The cases of 32 of these 179 patients had been reported previously^4^. The medical records of all 179 patients were reviewed to obtain data on clinical characteristics and the treatments that were received, and PCR data obtained from samples collected from the nasopharynx, sputum or saliva were collected. The study protocol was approved by the Ethics Committee of Keio University School of Medicine (approval number: 20200062) and was conducted according to the principles of the Declaration of Helsinki. This research is being conducted using an opt-out system (https://cmg.med.keio.ac.jp/covid19/) that discloses information about the conduct of the research without the need to obtain direct consent from the patient. The Ethics Committee of Keio University School of Medicine also approved that our study waived the need for informed consent.

### Definitions and classification of disease severity of COVID-19 infection

The disease severity of patients was classified according to the clinical management guidelines of the World Health Organization^30^ and Japan’s Ministry of Health, Labour, and Welfare (https://www.mhlw.go.jp/content/000650160.pdf). In some of patients with mild or moderate symptoms, the presence of pneumonia could not be determined because they did not undergo chest X-ray or computed tomography examinations. Therefore, we classified disease severity into the following three categories: “mild to moderate” (patients did not require supplementary oxygen); “severe” (patients required oxygen supplementation but not a ventilator); and “critical,” (patients who developed sepsis or acute respiratory distress syndrome and required a ventilator [Supplementary Table 2])^30^.

### DNA sequencing method

Whole viral genome sequencing, PCR-based amplification and phylogenic tree analysis were performed as previously reported (Takenouchi et al.)^4^. All point mutations including non-synonymous and synonymous mutations were annotated with ANNOVAR software and assessed with VarSifter (https://research.nhgri.nih.gov/software/VarSifter/). The multiple amino acid sequence alignments of various β-coronaviruses were compared using Molecular Evolutionary Genetic Analysis software (MEGA, https://www.megasoftware.net/) (Supplementary Table 3). The functional relevance of non-synonymous mutations was predicted with a Protein Variation Effect Analyzer (PROVEAN v1.1.3, http://provean.jcvi.org/seq_submit.php), the calculations of which are not dependent on sequence conservation among animals. Scores under a threshold value of − 2.50 were considered deleterious. We also used the software Phylogenetic Assignment of Named Global Outbreak Lineages (Pangolin; https://cov-lineages.org/index.html) to assign viral lineages in an automatic and precise manner^9^.

### Cloning and protein preparation of SARS-CoV-2 3CL^pro^

The SARS-CoV-2 3CL^pro^ DNA fragments encoding the Wuhan strain or the strain containing Pro108Ser in *non-structural polyprotein 5* (*NSP5)* gene were prepared using a reverse transcription kit (SuperScript III, ThermoFishher) and were amplified by PCR using primers (forward: 5′-TTTGGATCCAGTGGTTTTAGAAAAATGGCA-3′, reverse: 5′-TTTGTCGACTCATTGGAAAGTAACACCTGAGCA-3′). The fragments were digested with Bam HI and Sal I and then ligated into pCold GST containing the cleavage site for PreScision Protease (GE Healthcare) at the N-terminal region. The expression vectors for the 3CL^pro^ Wuhan strain type (WT) or the Pro108Ser mutant (P108S) were transformed into BL21 (DE3), and the bacteria were incubated in LB with ampicillin at 37 °C until OD600 was reached at 0.8. Protein expression was induced by 1 mM isopropyl-β-thiogalactopyranoside for 16 h at 4 °C. The cell pellets were re-suspended in a buffer containing 20 mmol/l Tris–HCl (pH7.5), 100 mmol/l NaCl, and 0.1% Tween 20, sonicated twice for 5 min at 4 °C, and centrifuged at 20,000×*g* for 30 min. The supernatant was incubated with glutathione Sepharose 4B (GE Healthcare) for 2 h at 4 °C. The resin was then washed five times with the same buffer, and the GST tag was cleared by the addition of PreScision Protease and further incubation for 16 h at 4 °C. Then, the 3CL^pro^ was prepared using size-exclusion chromatography (Superdex 200; GE Healthcare).

### Enzyme kinetics analysis using fluorescence resonance energy transfer-based assay

The enzymatic activities of 3CL^pro^ WT and P108S were determined using a fluorescent substrate with the cleavage site of SARS CoV-2 3CL^pro^ (Dabcyl-KTSAVLQ↓SGFRKME-Edans; GL Biochem). 3CL^pro^ WT or P108S at a final concentration of 5 μmol/l was incubated in a buffer of 20 m mol/l Tris-HCl (pH7.5), 100 m mol/l NaCl, and 5 m mol/l DTT with the addition of the substrate at a final concentration of 3.125, 6.25, 12.5, 25, 50, 100 or 200 μmol/l at room temperature. The change in fluorescence intensity was monitored with a fluorescence spectrophotometer (Cytation 5; BioTek) at an emission wavelength of 460 nm and an excitation wavelength at 340 nm. The kinetic parameters were determined with GraphPad Prism 8 software and the initial rate measurement of the substrate cleavage. For the inhibition assay, the SARS-CoV 3CL^pro^ inhibitor GC376 (Selleck) at a final concentration of 1, 2, 5, 10, or 20 μmol/l was incubated with 5 μmol/l 3CL^pro^ WT or P108S and 12.5, 25 or 50 μmol/l of substrate.

### Circular dichroism (CD) spectroscopy

CD spectra were collected in the far-UV (200–260 nm) and the near-UV (250–340 nm) spectral regions. Spectra were recorded with a CD spectrometer J-1500 (JASCO Corporation) in a quartz cuvette (1 mm cell length for far-UV and 10 mm for near-UV) at 20ºC. The protein samples were prepared in 20 mmol/l Tris–HCl buffer solution (pH 7.3) containing 150 mmol/l NaCl with concentration of 5 µmol/l for the far-UV CD measurements and 20 µmol/l for the near-UV CD measurements. The spectrum of the buffer was measured as a blank and was subtracted from the sample data. Four scans were averaged for each spectral region with a scan rate of 50 nm/min. The data pitch and the bandwidth were 0.5 nm and 1 nm, respectively.

### Sedimentation velocity analytical ultracentrifugation (SV-AUC)

The SV-AUC experiments were performed using the Optima AUC (Beckman Coulter) at 20 °C with 1, 2.5, 5, 10, 20, 40, and 80 µmol/l of 3CL^pro^ WT and P108S dissolved in 20 mmol/l Tris-HCl buffer solution (pH7.3) containing 150 mmol/l NaCl. Next, 390 µL of each sample was loaded into the sample sector of a 12-mm double-sector charcoal-filled Epon centerpiece, and 400 µL of buffer was loaded into the reference sector of each cell. Data collection was performed at 42,000 rpm using a UV detection system. Data were collected every 240 s with a radial increment of 10 µm at 230 nm for 1, 2.5, and 5 µmol/l samples, at 235 nm for 10 µmol/l samples, at 240 nm for 20 µmol/l samples, at 290 nm for 40 µmol/l samples, and at 295 nm for 80 µmol/l samples. The collected data were analyzed using a continuous *c*(*s*) distribution model implemented in program SEDFIT (version 16.2b) with fitting for the frictional ratio, meniscus, time-inmutation noise, and radial-inmutation noise. Both of the partial specific volumes of WT 3CL^pro^ and P108S were 0.731 cm^3^/g, which was calculated based on the amino acid composition of each sample using the program SEDNTERP 1.09. The buffer density and viscosity were calculated using the program SEDNTERP 1.09 as 1.00499 g/cm^3^ and 1.0214 cP, respectively. The *c* (*s*_20, w_) distribution figures were generated using the program GUSSI (version 1.3.2)^31^. The weight-average sedimentation coefficient of each sample was calculated by integrating the range of sedimentation coefficients where peaks with an obvious concentration dependence were observed. To determine the dissociation constant of the monomer–dimer equilibrium (KD), the concentration dependence of the weight-average sedimentation coefficient was fitted to the monomer–dimer self-association model implemented in the program SEDPHAT (version 15.2b)^32–34^.

### Hydrogen Deuterium Exchange Mass Spectrometry (HDX-MS)

HDX-MS experiments were conducted using Waters HDX with the LEAP system (Waters). Eighty μmol/l of protein solutions (SARS CoV-2 3CL^pro^ WT and SARS CoV-2 3CL^pro^ P108S) were diluted 20-fold with 20 mmol/l Tris–HCl buffer solution (pH 7.3) prepared with D_2_O containing 150 mmol/l NaCl, and incubated at 20 °C for various hydrogen/deuterium exchange time period (0.5, 1, 10, 60, or 240 min). The concentration of the protein solution during deuterium exchange was 4 μmol/l; and based on the *K*_D_ estimated from SV-AUC, each protein was considered to be present as more than 98% monomer. The exchange reaction was quenched by dropping the pH to 2.4 while mixing with an equal volume of 4 mol/l guanidinium chloride and 0.5 mol/l Tris (2-carboxyethyl) phosphine hydrochloride (TCEP) at pH2.2. One hundred picomoles of quenched samples were immediately injected, desalted, and separated online using a Waters UPLC system based on the nanoACQUITY platform. The online digestion was performed over 5 min in water containing 0.05% formic acid at 4 °C at a flow rate of 100 μL/min. The digested peptides were trapped on an ACQUITY UPLC BEH C18 1.7 μm peptide trap (Waters) maintained at 0 °C and desalted with water and 0.1% formic acid. Flow was diverted using a switching valve, and the trapped peptide fragments were eluted at 40 μL/min onto a column of 1 × 100 mm (C18 1.7 μm; ACQUITY UPLC BEH, Waters) held at 0 °C, with a 12-min linear acetonitrile gradient (8%–40%) containing 0.1% formic acid. The eluate was directed into a mass spectrometer (Synapt HD, Waters) with electrospray ionization and lock mass correction (using Glu-fibrinogen peptide B). Mass spectra were transformed using MassLynx (Waters) and acquired over the *m/z* range of 100–2000. Pepsin fragments were identified using a combination of exact mass and MS/MS, aided by ProteinLynx Global SERVER (PLGS, Waters). Peptide deuterium levels were determined using DynamX 3.0 (Waters). The relative deuterium uptake percentage was calculated for each peptide by dividing the mean of the deuterium uptake, $\overline{m}$, by the number of backbone amide hydrogens. In comparing the HDX results between two samples, the mass difference of hydrogen deuterium exchange for each peptide at each exposure time point (∆*HX*) was calculated as follows;

$$ \Delta HX = \overline{m}_{{sample_{1} }} - \overline{m}_{{sample_{2} }} $$

For the statistical analysis of significant difference, a volcano plot, which is a scatter-plot of Δ*HX* versus a probability value (*p* value) determined using the Welch *t*-test, was used^35^. The significance limits for the vertical $\Delta HX$ value were calculated as follows. A pooled sample standard deviation (*s*_*p*_) for 610 standard deviations was calculated from ∆*HX*. A propagated standard error of the mean (${\text{SEM}}_{\Delta HX}$) was calculated from *s*_*p*_. A significance limit for the ∆*HX* values can be calculated using the following equation;

$$ \left| {\Delta HX} \right| > k \times {\text{SEM}}_{\Delta HX} $$

We set *k* = 4.60 using a Student’s *t*-distribution value for a two-tailed test with four degrees of freedom at a significance level (α) of 0.01 (99% confidence level). For the horizontal *p* value, the significance limits were defined at α = 0.01 (99% confidence level).

### Statistical analysis

The main parameter of the clinical study was the grade of disease severity. Comparisons of categorical variables between the two groups were assessed using the Fisher exact test. A Student *t*-test was used to compare abnormally distributed quantitative variables between the two groups. An exact logistic regression analysis was used to examine the odds ratio for requiring supplemental oxygen. The following covariates were considered for inclusion in the multivariate model: age group (< 65 years or ≧ 65 years), sex (male or female), Charlson Comorbidity Index group (0 or ≧ 1), temporal trend (March to October 2020 or November 2020 to January 2021) and infection group (Clade 20B-T or 20B-nonT). Statistical analyses were performed using R statistical Software (version 3.6.2), and all the statistical tests were two-sided. A *p* values of < 0.05 was considered significant.

## Supplementary Information

Supplementary Information.

## Data Availability

The authors declare that the data supporting the findings of this study are available within the article, Supplemental Data, and Supplemental Method files. Source data are provided with this paper. We downloaded the full nucleotide sequences of the SARS-CoV-2 genomes from the GISAID database (https://www.gisaid.org/). A table of the contributors is available (acknowledgment table). We have uploaded the full nucleotide sequences of our cohort to the GISAID database.
