# Supplementary material for: Pro108Ser mutation of SARS-CoV-2 3CLpro reduces the enzyme activity and ameliorates the clinical severity of COVID-19
Source: Sci Rep. 2022 Jan 25;12:1299. doi: 10.1038/s41598-022-05424-3 (PMC8789791; doi:10.1038/s41598-022-05424-3)
Supplement: Supplementary file 1 — Supplementary Information. [file 41598_2022_5424_MOESM1_ESM.docx]

# ***Supplementary Table 1:* Clinical backgrounds of COVID-19 included 179 patients**

|  | N= 179 |
| --- | --- |
| Mean age (years old) | 45 ± 19 |
| Sex (male / female) | 118 / 61 |
| Symptoms at admission |  |
| Cough | 86 (48.0 %) |
| Dysosmia | 26 (14.5 %) |
| dysgeusia | 27 (15.1 %) |
| Fever (≧ 37.5°C) | 101 (56.4 %) |
| Sepsis | 2 (1.1 %) |
| Acute respiratory distress syndrome | 15 (8.4 %) |
| Treatment |  |
| Oxygen administration | 45 (25.1 %) |
| Methylprednisolone treatment | 35 (19.6 %) |
| Ventilator | 12 (6.7 %) |
| Intensive care unit admission | 16 (8.9 %) |
| Death | 7 (3.9 %) |
| COVID-19, coronavirus disease 2019. | |

# ***Supplementary Table 2:* Severity of COVID-19 patients**

| Grade | Definition | Total number |
| --- | --- | --- |
| Critical | Patients who developed sepsis or acute respiratory distress syndrome with need for ventilators | 16 |
| Severe | Patients who needed oxygen administration with no need for ventilators | 29 |
| Mild-Moderate | Patients who did not need oxygen administration | 134 |
| COVID-19, coronavirus disease 2019. | | |

# ***Supplementary Table 3:* The sequences of strain used as a reference genome**

| Strain | Complete genome | ORF 1ab polyprotein | Nucleocapsid protein |
| --- | --- | --- | --- |
| COVID-19 Wuhan | NC_045512.2 | YP_009724389.1 | YP_009724397.2 |
| SARS-Coronavirus BJ01 | AY278488 | AAP30028.1 | AAP30037.1 |
| SARS-related Coronavirus | DQ898174 | ABI96956.1 | ABI96968.1 |
| SARS-Coronavirus Tor2 | NC_004718 | NP_828849.7 | YP_009825061.1 |
| MERS-Coronavirus | NC_019843 | YP_009047202.1 | YP_009047211.1 |
| Human β-coronavirus EMC | JX869059 | AFS88944.1 | AFS88943.1 |
| Human β-coronavirus Jordan-N3 | KC776174 | AGH58716.1 | AGH58724.1 |
| Human enteric coronavirus 4408 | FJ415324 | ACJ35483.1 | ACJ35489.1 |
| COVID-19, coronavirus disease 2019; SARS, severe acute respiratory syndrome, MERS, middle-east respiratory syndrome; 3C-like, 3 chymotrypsin-like; ORF, open reading frame. | | | |

# ***Supplementary Fig. 1* Temporal trends of haplotypes with Pro108Ser mutation in 3CL^pro^ and Pro151Leu in nucleocapsid protein among Clade 20B in Japan.**

A magnified view of the dotted square obtained from Fig. 3a. showed that Pro151Leu mutation in nucleocapsid protein was emerged in April 2020 earlier than Pro108Ser mutation in 3CL^pro^ (May 2020).

# ***Supplementary Fig. 2* The evaluation of chirality between WT and P108S using CD spectroscopy.**


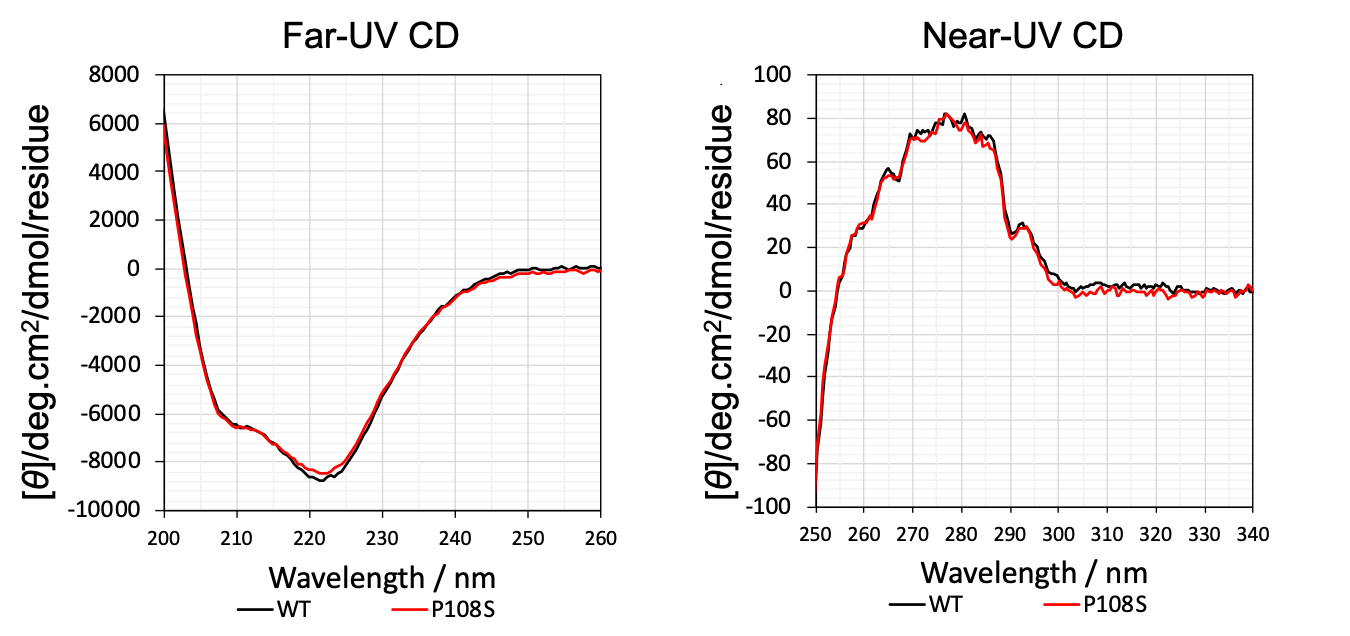


CD spectra were collected in the far-UV (200-260 nm) and the near-UV (250-340 nm) spectral regions. CD, Circular dichroism; UV, ultraviolet; WT, Wuhan-strain type; P108S, Pro108Ser mutant.

# ***Supplementary Fig. 3* Flowchart of COVID-19 patients.**

This study finally included 179 out of 311 COVID-19 patients treated during the study period. COVID-19, coronavirus disease 2019; PCR, polymerase chain reaction.

***Supplementary Fig. 4* Full-size SDS-PAGE of recombinant WT or P108S of SARS-CoV2 3CL^pro^**

***
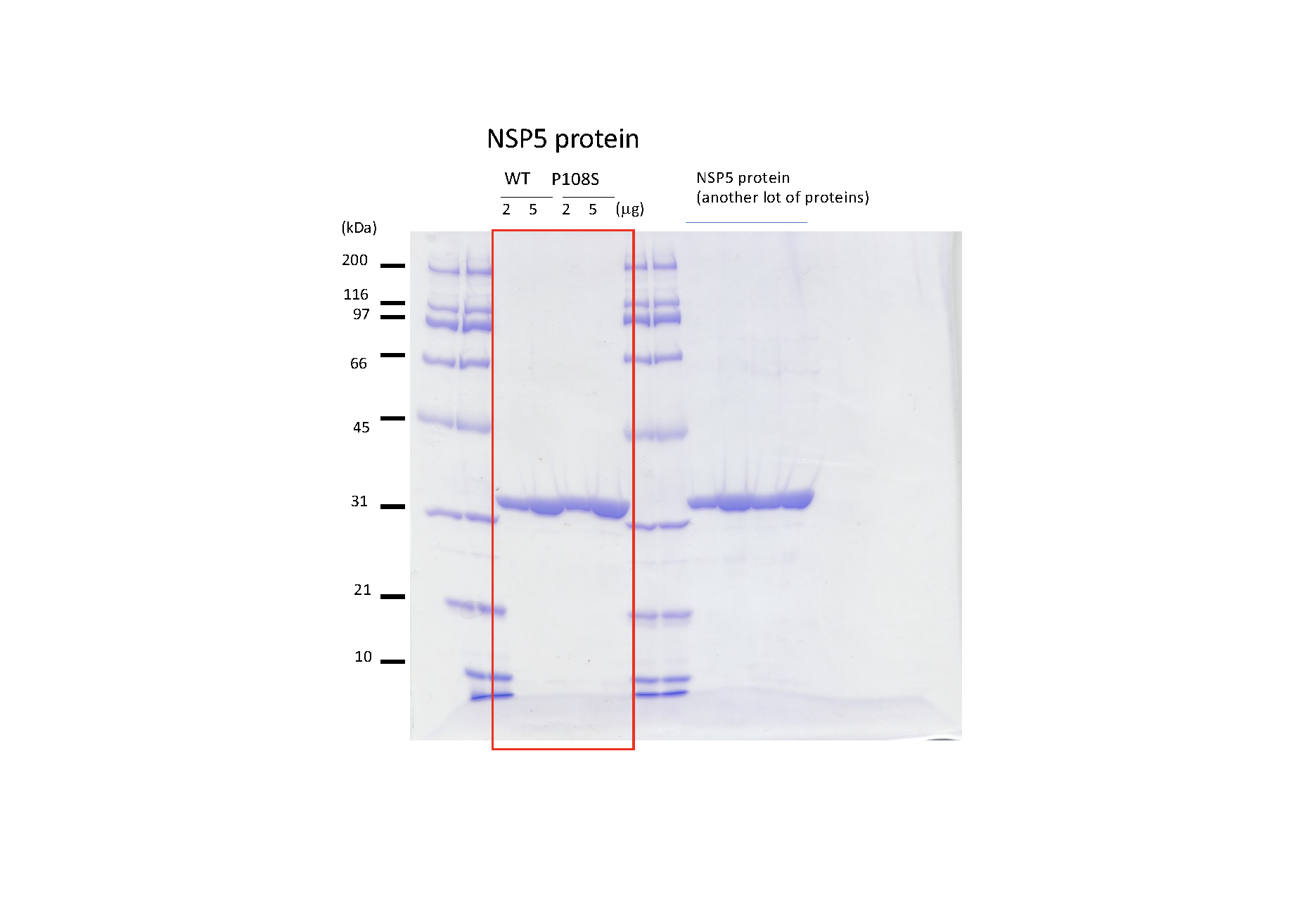
***

This figure was the full image of Fig.4a.
